# Blood derivatives as monotherapy and combination therapy: A promising strategy for wound healing

**DOI:** 10.1016/j.reth.2025.10.018

**Published:** 2025-11-01

**Authors:** Majid Zamani, Mohammad Masumzadeh, Mohammadreza Mohammadi Hosn, Fatemeh Pouladkhay

**Affiliations:** a Department of Hematology, Faculty of Medical Sciences, Tarbiat Modares University, Tehran, Iran; b Department of Anesthesia, School of Paramedical Sciences, Gonabad University of Medical Sciences, Gonabad, Iran; c Department of Orthopedic, School of Medicine, Golestan University of Medical Sciences, Gorgan, Iran; d Department of Operating Room, School of Paramedical Sciences, Gonabad University of Medical Sciences, Gonabad, Iran; e National Center for Health Insurance Research, Tehran, Iran

**Keywords:** Regenerative medicine, Wound healing, Growth factors, Blood derivatives, Combination therapy

## Abstract

Wound healing is a highly orchestrated biological process, and any disruption or delay in its progression can result in the formation of chronic wounds. Such conditions impose a considerable clinical and socioeconomic burden on both patients and healthcare systems. Over the years, numerous therapeutic strategies have been investigated to promote tissue repair, with varying degrees of success. Among these, blood-derived products have emerged as a focal point of interest, owing to their regenerative potential and bioactive composition. Preparations including platelet-rich plasma, platelet-rich fibrin, platelet lysates, and autologous conditioned serum have demonstrated the capacity to enhance healing through the delivery of concentrated growth factors and cytokines. Each derivative possesses distinct advantages and limitations determined by its cellular content, biomolecular profile, and method of preparation. Most commonly presented in liquid or gel form, these products can be applied to diverse wound types and tailored to specific treatment protocols. Their use may be autologous or allogeneic, and they can be employed alone or in combination with other therapeutic modalities to achieve synergistic effects. This review provides a comprehensive overview of the characteristics, preparation techniques, biochemical composition, and clinical efficacy of various blood derivatives, underscoring their value either as standalone interventions or as part of multimodal regimens in advancing wound healing.

## Highlights

• Each blood derivative has unique biological properties.
• Blood derivatives are effective in promoting wound healing.
• Blood derivatives show enhanced efficacy when used in combination with other treatments.

## 1 Introduction

Wound healing is a complex mechanism, and any delay can lead to wound chronicity. Chronic wounds impose significant psychological and financial burdens on patients, as well as substantial costs and challenges for the healthcare system [1]. The goal of various wound treatments is to accelerate and promote healing. Different treatments are used for acute and chronic wounds [2]. Appropriate treatment for acute wounds can result in faster healing and prevent scarring. In chronic wounds, such as diabetic foot ulcers where angiogenesis is impaired, treatments can help by promoting angiogenesis [3]. One such treatment is the use of blood derivatives, which, due to the presence of growth factors and various cytokines, can aid in the proliferation, differentiation, and migration of cells at the wound site, providing a suitable environment for cell growth and promoting healing [4,5]. Various blood derivatives with different characteristics have been used for wound healing. Products such as platelet-rich plasma (PRP) are obtained by centrifuging blood containing anticoagulant and separating plasma along with platelets and leukocytes. These products contain various blood cells, including platelets, which play a key role in wound healing [6]. Platelet-rich fibrin (PRF) is an easily prepared product in gel form, containing blood cells and no anticoagulant [7,8]. Platelet lysate (PL) is obtained from the destruction of platelet membranes and contains platelet-derived growth factors but is cell-free [9]. Additionally, autologous conditioned serum (ACS) is obtained from the activation of white blood cells, especially monocytes, by glass beads and contains high levels of anti-inflammatory cytokines that help regulate inflammation [10]. The aim of this study is to review wound healing, the various blood derivatives used in wound healing, the characteristics of these blood derivatives and the growth factors derived from them, as well as the effectiveness of both standalone and combination treatments involving blood derivatives.

## 2 Blood derivatives

The use of blood derivatives has attracted significant attention in recent decades. Blood derivatives have been applied in various fields of medicine [11]. They come in different types based on their preparation methods and the components they contain. The characteristics and compounds present in each blood derivative make it suitable for specific applications. Some blood derivatives, such as PRP, contain blood cells including platelets, while others, such as PL and ACS, are cell-free and contain compounds released from cells [5,11,12]. All of these blood derivatives have been widely used in regenerative medicine, especially in wound healing [12,13]. The characteristics of each blood derivative are summarized in Table 1.

**Table 1:** Characteristics of various blood derivatives.

Feature	PRP	PRF	PL	ACS
Anticoagulant	+	–	+	–
Contains blood cells	+	+	–	–
Liquid form	+	–	+	+
Gel form	+	+	+	–
Production cost	++	+	+++	++++
Studies on wound healing	High	High	High	low
Centrifuge step	1–2	1	2–3	1
Filtration	–	–	0.22 μm	0.22 μm
Incubation	–	–	–	+
Time-consuming production	++	+	++++	++++
Special advantage	First generation, Easy to prepare with platelet concentration capability	Slow release of growth factors, No anticoagulant	High concenteration of growth factors	High concenteration of anti-infelimatory cytokine

### 2.1 Platelet-rich plasma

Platelets are important cells in hemostasis and thrombosis. They play a role in tissue regeneration, angiogenesis, and tissue repair, as well as in regulating the balance between programmed cell death and the survival of cells involved in tissue repair [14,15]. Platelets contain a wide range of growth factors and chemokines, which are mostly released from platelet alpha granules. Platelet growth factors and chemokines include: vascular endothelial growth factor (VEGF), transforming growth factor-beta (TGF-β), epidermal growth factor (EGF), insulin-like growth factor-1 (IGF-1), platelet-derived growth factor (PDGF), basic fibroblast growth factor (b-FGF), hepatocyte growth factor (HGF), tumor necrosis factor-alpha (TNF-α), gamma interferon (IFN-γ), granulocyte-macrophage colony-stimulating factor (GM-CSF), granulocyte colony-stimulating factor (G-CSF), interleukin-8 (IL-8), IL-7, IL-6, IL-1α, chemokine ligand 1 (CXCL-1), CXCL-4 (PF4), CXCL-5, CXCL-2, CXCL-3, CXCL-10, all of which play roles in cell differentiation and proliferation, angiogenesis, and wound healing [[16], [17], [18], [19]]. Growth factors secreted from blood cells, especially TGF-β, PDGF, b-FGF, IGF, VEGF, EGF, and EGF family members (including TGF-α and heparin-binding EGF-like growth factor (HB-EGF)), play a critical role in wound healing by stimulating the proliferation and migration of fibroblasts, keratinocytes, and vascular and endothelial cells to the wound site, thereby increasing re-epithelialization, angiogenesis, and collagen deposition to promote tissue regeneration and wound healing [[20], [21], [22], [23], [24], [25]]. The function of major growth factors in wound healing is summarized in Table 2. The advantages of PRP include its liquid form, which allows it to be used in combination with other treatments, and its ability to be converted into a gel by adding compounds such as calcium chloride, calcium gluconate, or thrombin [[26], [27], [28]].

**Table 2:** Important growth factors in wound healing [5,19,[21], [22], [23], [24], [25],^57^,^59^].

Growth Factors	Source	Function
Transforming growth factor beta (TGF-β)	Platelets, keratinocytes, macrophages, lymphocytes, fibroblasts	Regeneration of connective tissue, Cell differentiation, prolifereation, migration, granulation tissue formation, re-epithelialization, angiogenesis, inflammation, matrix formation and remodeling, collagen synthesis and disposition, Important in tissue remodeling and scar formation
basic-fibroblast growth factor (b-FGF)	Keratinocytes, mast cells, fibroblasts, endothelial cells, smooth muscle cells, chondrocytes	Cell differentiation, prolifereation, migration, granulation tissue formation, re-epithelialization, angiogenesis, matrix formation and remodeling, collagen synthesis
Epidermal growth factor (EGF)	Platelets, macrophages, fibroblasts	Keratinocyte cells mitosis and migration, re-epithelialization, angiogenesis, wound tensile strength, and reduced healing time of venous ulcer and diabetic foot ulcer
Transforming growth factor alpha (TGF-α)	Macrophages and keratinocytes	Binding to the same receptor as EGF, cell proliferation, growth, survival, and differentiation, tissue regeneration, wound healing, embryogenesis
Heparin-binding EGF-like growth factor (HB-EGF)	Macrophages and keratinocytes	Cell prolifereation and migration, increase re-epitalization, collagen content, wound healing, and tissue regeneration.
Platelet-derived growth factor (PDGF)	Platelets, keratinocytes, macrophages, endothelial cells, fibroblasts	Cell prolifereation, migration, development of neurons, mitogen, granulation tissue formation, re-epithelialization, angiogenesis, chemotactic, inflammation, matrix formation and remodeling, essential in the early stages of wound healing
Insulin-like growth factor (IGF)	Liver, platelet, fibroblasts, and other tissues	Cell differentiation, prolifereation, migration, angiogenesis, neuroporotective, re-myelination, production of collagen type II, stimulate synthesis of fatty acid, tissue repair and regeneration
Vascular endothelial growth factor (VEGF)	Platelets, macrophages, fibroblasts, endothelial, smooth muscle cells, neutrophils	Cell differentiation, prolifereation, migration, development of neurons, mitogen, granulation tissue formation, angiogenesis, chemotactic, vascularization, neurogenesis, neuroporotective,

#### 2.1.1 PRP preparation method

**Fig. 1:**
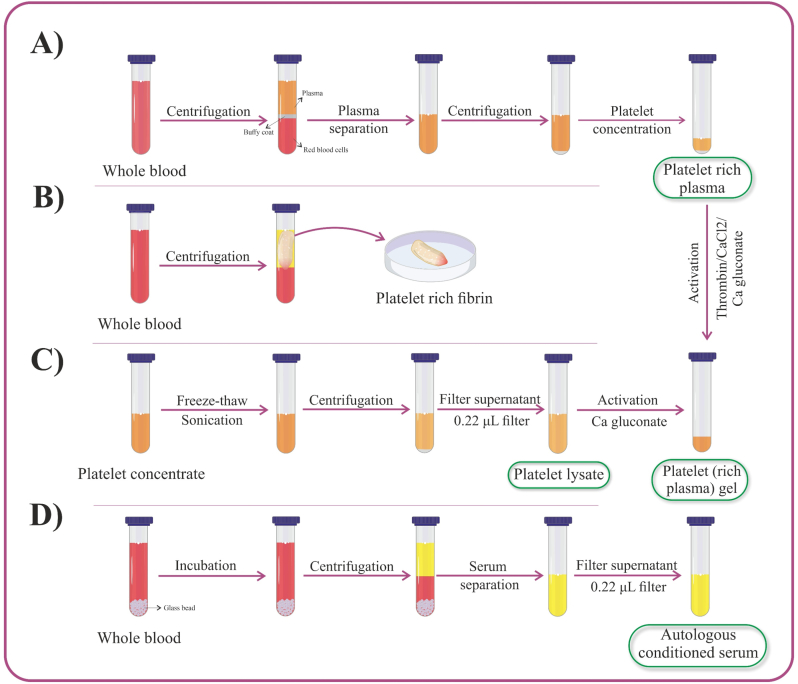
**Methods for preparing various blood derivatives. A)** To prepare platelet-rich plasma, blood is collected in a tube containing anticoagulant and centrifuged. The platelet-rich plasma is separated and concentrated. Activators may be added to form a gel. **B)** To prepare platelet-rich fibrin, blood without anticoagulant is centrifuged, and the fibrin clot is collected. **C)** To prepare platelet lysate, the platelet membranes in PRP or platelet concentrate are disrupted via freeze–thaw cycles or sonication. The product is then centrifuged and filtered using a 0.22 μm filter. **D)** To prepare autologous conditioned serum, blood is incubated in a container with glass beads, followed by centrifugation. The serum is then separated and filtered using a 0.22 μm filter.

There are different methods for preparing PRP. Two-stage centrifugation is considered a more suitable method for PRP preparation [29]. Methods for preparing different types of blood derivatives are shown in Fig. 1. After blood collection in an anticoagulant tube, the sample is first centrifuged at a low speed. After centrifugation, three layers are formed: red blood cells, buffy coat (containing platelets and leukocytes), and plasma. The plasma and buffy coat layers are separated and centrifuged again at a higher speed. After this step, platelets and leukocytes precipitate, and platelet-poor plasma (PPP) remains on top. By removing more than half of the plasma volume, the remaining sample is gently agitated to prepare PRP [19]. Finally, the sample can be gelled by adding calcium chloride (CaCl_2_), calcium gluconate, or thrombin [[26], [27], [28]] (Fig. 1A).

### 2.2 Platelet-rich fibrin

Although PRP was widely used as the first blood derivative, in recent years, a second-generation blood derivative, PRF, has been developed with the primary goal of eliminating anticoagulants. PRF has a greater capacity for the sustained release of growth factors and has more clinical applications [30]. The advantage of PRF over PRP is the slower release of cell growth factors. PRF contains autologous cells, along with growth factors, entrapped within a fibrin mesh and is slowly released at the site of application [30].

#### 2.2.1 PRF preparation method

There are various methods for preparing PRF. In general, the principles of these methods are similar, but changes in tube material, centrifugation time, and speed affect the amount of leukocytes, platelets, and growth factors in the sample [31]. To prepare PRF, after blood collection in a tube without anticoagulant, the tube is immediately centrifuged. After centrifugation, the red blood cells settle to the bottom layer, and the platelets and leukocytes are trapped in the fibrin clot. The PRF clot is then separated from the red blood cells and can be used directly or further processed, depending on the clinical application. [32]. Methods for preparing PRF are shown in Fig. 1B.

### 2.3 Platelet lysate

PL is obtained by activating or disrupting the cell membranes of platelets. Upon activation, platelets release various growth factors and cytokines into the plasma, which are also present in PL [33]. PL contains higher concentrations of growth factors than PRP and can be stored in the freezer for extended periods. In addition to being cost-effective and easy to prepare, PL may serve as a suitable alternative to PRP due to the removal of platelet membrane debris and leukocytes [18]. PL has been used to promote the proliferation of various cell types, and its effects are largely attributed to its growth factor content. The abundance of these bioactive molecules has increased interest in using PL in cell culture and regenerative medicine [17].

#### 2.3.1 PL preparation method

PL can be produced through various methods, including freeze-thaw cycles and ultrasonication. Methods for preparing PL are illustrated in Fig. 1C. To prepare PL, a platelet concentrate must first be obtained, typically through platelet apheresis or PRP preparation, as previously described. Once the concentrate is prepared, it can be activated and disrupted using the aforementioned techniques. The freeze-thaw method is generally preferred, as it does not require the addition of exogenous compounds, thereby avoiding potential adverse effects. Typically, 3–5 cycles of freezing at −80 °C and thawing at 37 °C are employed [5]. To remove cell debris, the product is filtered with a 0.022 μm filter. Finally, the sample can be gelled by adding calcium gluconate [34]. One advantage of PL is its use as a supplement in cell culture media, potentially replacing fetal bovine serum (FBS). To prevent clotting in calcium-containing media, heparin may be added [17,18].

### 2.4 Autologous conditioned serum

ACS is a topical source of the anti-inflammatory cytokine interleukin-1 receptor antagonist (IL-1Ra). The development of ACS was based on studies showing that macrophages and monocytes are endogenous sources of IL-1Ra [35]. This cytokine can be produced by activating these immune cells through contact with various surfaces. Accordingly, Meijer et al. [36] investigated the effectiveness of glass beads for ACS production. Using a syringe filled with glass beads, they facilitated contact between monocytes and other adherent peripheral blood cells. Their findings showed increased production of cytokines, particularly IL-1Ra. Due to its high IL-1Ra concentration, ACS has been widely used in studies targeting knee osteoarthritis [35].

#### 2.4.1 ACS preparation method

To prepare ACS, blood is collected in a glass bead-containing tube without anticoagulants and incubated for 6–24 h. The sample is then centrifuged, and the serum is separated. To remove cell debris, the product is filtered with a 0.022 μm filter [12,35]. The method is depicted in Fig. 1D.

## 3 Blood derivatives in wound healing

**Fig. 2:**
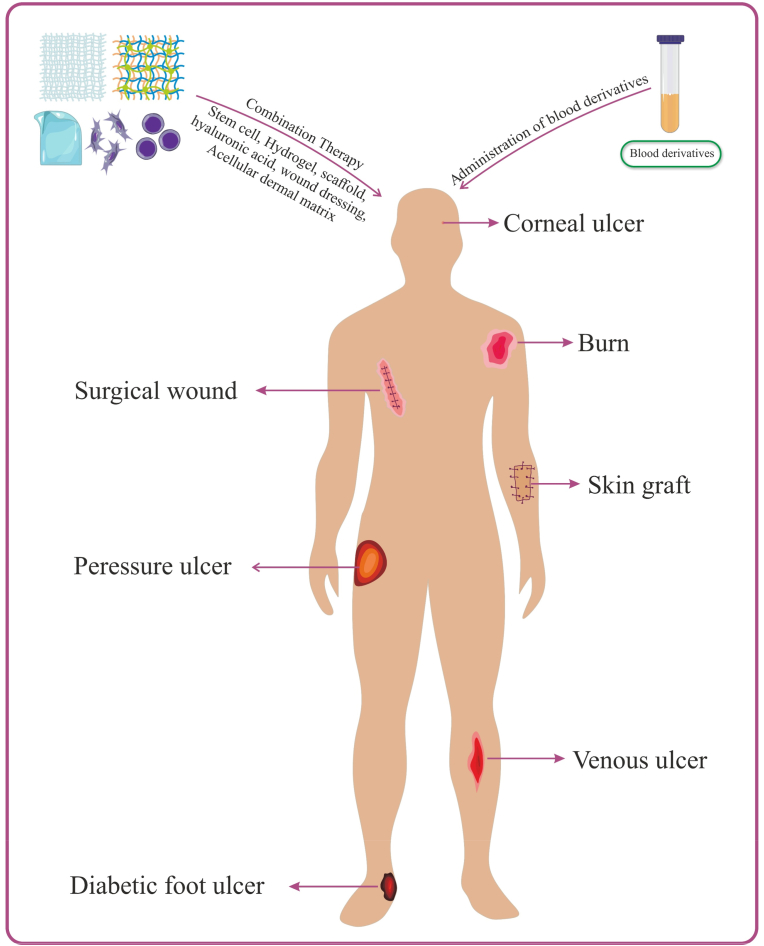
**Applications of blood derivatives in wound healing.** Blood derivatives can be used alone or in combination with other therapies to treat various types of wounds.

Various studies have investigated the use of blood derivatives such as PRP, PL, and ACS in wound healing. Their applications are illustrated in Fig. 2, and the results of studies assessing their effectiveness are summarized in Table 3. These derivatives can support cell proliferation and tissue repair by providing an array of growth factors, cytokines, and micronutrients [11]. One advantage of plasma-based derivatives is their applicability in gel form. For example, PRF provides a sustained release of growth factors at the wound site, resulting in prolonged therapeutic effects [6]. PL offers high growth factor content and lacks cellular elements, although cellular components may also play a role in modulating treatment responses [5]. ACS is distinguished by its high concentrations of anti-inflammatory cytokines and the absence of cell membranes [35]. While many studies have demonstrated the efficacy of blood derivatives in wound healing, some have reported limited or no effects [37]. Factors such as preparation methods and platelet concentration significantly influence product quality and therapeutic outcomes. Moreover, wound healing is a complex, multifactorial process affected by wound type and the patient's overall condition. A study revealed that different concentrations of blood derivatives can yield varying outcomes in wound repair [38]. Higher concentrations of PL were associated with improved healing. In an animal model, three PL formulations, cryopreserved PL, cryopreserved and lyophilized PL, and lyophilized and refrigerated PL, were compared, with no significant differences observed in wound healing efficacy [39]. Scevola et al. [40] examined the use of platelet gel in pressure sores and found it beneficial during the initial two weeks of treatment, though less effective in the long term for chronic wounds. Diab et al. [6] compared PRP and PRF in treating atrophic acne scars, reporting greater efficacy and patient satisfaction with PRF, despite similar outcomes in scar appearance. Alhawari et al. [41] demonstrated that PL was more effective than PPP in treating diabetic foot ulcers, likely due to the higher growth factor concentration in PL and the lower platelet count in PPP. Another advantage of blood derivatives is their potential for allogeneic use. Studies have shown that allogeneic blood derivatives can be effective and comparable to autologous counterparts [[42], [43], [44], [45]]. This is particularly valuable for patients who are unable to provide suitable autologous blood. However, appropriate testing and screening for transmissible diseases are essential.

**Table 3:** Summary of studies on the effects of blood derivatives and their effectiveness in wound healing.

Blood derivative	Subject	Application	Wound type	Outcome	Anticoagulant	Centeriguge	Activator	Reference
PRP^a^	Mice	–	6 mm full-thickness cutaneous wound	Increase migration, survival and proliferation of primary cultured Embryonic Stem Cells and differentiation into adult cells (keratin 10, keratin 14, and CD49f), Regulate local inflammation, increase angiogenesis and re-epithelialization, local vessel intensity, increase VEGF and IGF-1 secretion, reduce IL-17 and IL-1β production (inflammatory cytokines)	Heparin sodium	300×*g*, 10 min/300×*g*, 20 min	Freeze–thaw	[64]
PRP	Diabetic rat	Allogeneic	1.5 cm full-thickness cutaneous wound	Accelerated wound healing, in the granulation tissue of ulcer wounds expression levels of inflammatory cytokines IL-10, IL-1β, *NLRP3*, and MDA content was lower, SOD content was higher, and expression levels of *GPX4*, *SLC7A11, CD31* and *VEGF was higher and ACSL4* was lower, inhibit ferroptosis effect, increase the migration and regeneration ability of fibroblasts and vascular endothelial cells induced by high glucose	Sodium citrate	200 g for 10 min/2500 g for 15 min 2000 g for 10 min for remove cell debris 0.22-pm	Calcium gluconate, bovine thrombin	[66]
PRP	Rabbit	Allogeneic	Corneal ulcer	Improve noninfectious extensive corneal ulcers	sodium citrate	1000 RPM for 5min	–	[67]
PRP	Hman	Autologous	Chronic venous leg ulcers	Reduce ulcer size and pain	Citrate dextrose	277 g for 10 min/277 g for 5 min	Ca gluconate	[68]
PRP	Human	Autologous	Surgery wound in the maxillofacial region	Promote wound healing, fibroblasts, macrophages, and collagen fibres, higher concenteration of IL-1β and TNFα in wound fluid and improve inflammatory and granulation phases	Sodium heparin	3000 rpm for 5 min	–	[69]
PRP	Hman	Autologous	Trophic Ulcers in Leprosy	Reduce ulcer size and time of ulcer healing, increase re-epithelization	Acid citrate dextrose	2000 rpm for 10 min/3000 rpm for 10 min	Calcium chloride	[70]
PRP	Human	Autologous/allogeneic	Diabetic lower extremity ulcers	Wound healing time and adverse reactions were similar in both allogeneic and autologous groups.	Acid citrate dextrose solution B	600 rpm for 15 min/1135 g for 7 min	Bovine thrombin, calcium gluconate	[45]
PRP	Human	Allogeneic	Chronic wound	Indicate bright red granulation which bled easily, accelerate wound healing, decrease inflammation, no rejection reactions	Sodium citrate	400g for 10 min/1200g for 20 min	Calcium gluconate	[71]
PRP	Human	Autologous	Corneal ulcer	Some clinical improvements in refractory corneal ulcers	Citrate Phosphate Dextrose Adenine-1	800 rpm for 10min	–	[72]
PRP	Human	Autologous	Dormant corneal ulcer	Better healing outcome	Sodium citrate	-/10min	–	[73]
Conditioned Plasma	Rat	Xenograft (Plasma was for human)	8 mm full-thickness excisional wound	Promote wound healing, enhanc fibroblasts proliferation, epithelization, synthesis of collagen fibers and reduce inflammatory cells infilteration	Citrate dextrose	For PRP: 10 min at 160 g and 15 min at 400 g For conditioned plasma: incubated for 6 h, 10 min at 400 g	Polylactic acid coated glass beads for conditioned plasma	[20]
PRP gel	Human	Autologous	Chronic wound/diabetic, pressure, or venous ulcer; dehisced, surgical, or traumatic wound; and wounds of other etiologies	Reduce wound area	–	–	–	[74]
PRP gel	Human	Autologous	Acute traumaticwounds	Increase wound healing rate	Acid citrate dextrose solution A	3200 rpm for 20 min	Thrombin	[26]
Platelet gel	Human	Allogeneic	Pressure sores	Can improve wound healing in the first two weeks	–	–	Calcium chloride	[40]
PRP gel	Human	Autologous	Diabetic chronic refractory cutaneous ulcers	Improves the wound healing grades, shortens the healing time and accelerates the healing velocity.	–	313 g for 4 min/1252 g for 6 min	Thrombin and calcium gluconate	[27]
PRP gel	Human	Autologous	Diabetic Foot Ulcers	Accelerate wound healing	–	1500 rpm for 5 min/3500 rpm for 5 min	Thrombin	[75]
PRP gel	Human	Autologous	Diabetic Foot Ulcer	Increase wound closure and healing	Sodium citrate	2000 g for 10 min	Calcium chloride	[28]
Platelet gel	Human	Allogeneic	Chronic wound	Promote wound healing	–	1500 g for 8 min	Thrombin, Calcium chloride, aprotinin, fibrinogen, coagulation factor XIII	[44]
PRP/PPP^b^	Human	Autologous	Diabetic foot ulcers	Increase wound healing	Citrate dextrose	1007 g/447.5 g	Thrombin, Calcium chloride	[76]
PRP/PRF^c^	Human	Autologous	Atrophic acne scars	Acne scars improve was same in both PRP and PRF, According to quartile grading scale and patient satisfaction; the therapeutic response was significantly higher in PRF group than PRP	Ethylenediaminetetraacetic acid for PRP	For PRP: 900 rpm for 5 min/2000 rpm for 15 min For PRF: 700 rpm for 3 min	Calcium chloride	[6]
PRF	Rat	Allogeneic	Ischaemia/reperfusion injury	Accelerated skin wound healing, increase angiogenesis, and no increase in fibrotic tissues	–	400g for 10 min	–	[42]
PRF	Cat	Autologous	2 cm^2^ full thickness cutaneous wound	Promote healing	–	3500 rpm for 10 min	–	[7]
PRF	Feline	–	15 mm full-thickness cutaneous wound	Accelerated skin wound healing, reducing inflammation, increase synthesis of growth factors increase formation of collagen fibers	–	400 g for 10 min	–	[8]
PRF	Human	Autologous	Total Laryngectomy	Accelerate the wound healing, reduce pain and edema, and activate leucocytes	–	3000 rpm (400 g) for 10 min	–	[77]
Leukocyte-and platelet-rich fibrin	Human	Autologous	Chronic wounds: venous leg ulcer, diabetic foot ulcer, pressure ulcer, or complex wounds	Reduce wound area	–	2700 rpm (400 g) for 12 min	–	[78]
								
PRF	Human/micrograft spray-on skin	Autologous	Flame burn/hydrochloride acid burn	In two cases of massive burns, rapid re-epithelialization was reported. Additionally, while skin grafting failed in two chronic burn patients, one such patient achieved complete wound healing within a week.	–	Ultracentrifugation for 25 min	–	[79]
PRF	Human	Autologous	Diabetic foot ulcer	Improved wound healing	–	400 g, 12 min	–	[80]
PRF	Human	Autologous	Chronic wound	Promote wound closure and healing	–	3000 RPM (805 g), 10 min	–	[81]
PL^d^	Rat	Allogeneic	0.12–0.3 cm^2^ full-thickness excisional wound	Promoting epithelialization and granulation tissue formation	–	–	–	[43]
PL	Human	Autologous	Chronic venous ulceration	No significant differences in wound healing were observed between the treatment groups	Ethylenediaminetetraacetic acid	500 rpm for 70 min/3000 rpm for 10 min 3000 rpm for 10 min centrifuge at 4000 rpm for remove Platelet fragments	Sonication for 25 s in 5 s bursts	[37]
PL/PPP	Human	Autologous	Diabetic foot ulcers	Accelerate and promote wound healing, PL superior to PPP	Citrate	For PL: 900 g for 10 min 3060 g for 20 min for remove derbies and filtered the supernatant through a 0.22 μL filter For PPP: 3060 g for 20 min and filtered the supernatant through a 0.22 μL filter	–	[41]
Umbilical cord blood -PL	Human	Allogeneic	Diabetic foot ulcers	Reduce ulcer size	–	865 g for 15 min/2500 g for 15 min	Freeze-thaw/−80/37 °C 48h calcium gluconate	[34]
PL	Human	Autologous	Diabetic foot ulcers	Promote wound closure, epidermal keratinocytes migration	Citrate	1800 g for remove derbies and filtered the supernatant through a 0.22 μL filter	Freeze-thaw/−80 2time	[9]
ACS^e^	Diabetic mice	Autologous	10 mm full-thickness excisional wound	Promote wound healing, enhanc fibroblasts, inactivate STING signaling pathway	–	15 min at 1500 rpm incubated for 6 h	Glass beads	[65]

a Platelet rich plasma.

b Platelet poor plasma.

c Platelet rich fibrin.

d Platelet lysate.

e Autologous conditioned serum.

### 3.1 Combination therapies with blood derivatives

Blood derivatives can be used in combination with other therapies, enhancing their efficacy [46]. Related studies are summarized in Table 4. For example, combining blood derivatives with stem cells improves the local environment for cell proliferation and tissue regeneration. [47]. When combined with hydrogels, blood derivatives provide a conducive environment for wound healing and allow for controlled release of growth factors [[48], [49], [50]]. Combination with wound dressings enhances moisture retention, improves healing conditions, and supplies essential biomolecules [12]. Though many studies support the efficacy of such combination therapies, some have reported limited benefits [51]. As previously discussed, the effectiveness of blood derivatives depends on product quality, patient condition, and the wound's nature. Additionally, the type of therapy or cell used may influence treatment outcomes. Animal and human studies have demonstrated the effectiveness of combining allogeneic blood derivatives with other therapies [3,4,52,53].

**Table 4:** Summary of studies on the effects of combination therapy using blood derivatives in wound healing.

Blood derivative	Combination	Subject	Application	Wound type	Outcome	Anticoagulant	Centeriguge	Activator	Reference
PRP^a^	MSCs	Diabetic mice	MSCs Autologous/PRP Allogeneic	15 mm full-thickness cutaneous wound	Re-epithelialization was not significantly different in the MSCs group alone and in combination with PRP.	Sodium citrate	600 g for 5 min	Sodium gluconate	[51]
PRP	Acellular dermal matrix	Mice	Xenograft (PRP was for human)	1.2 cm full-thickness cutaneous wound	ADM/PRP combination promote revascularization, rapid epithelialization and well-differentiated epidermis, and earlier collagen development and regularly arrengment	–	2060g for 18 min/2060*g* for 20 min	–	[82]
PRP	Adipose-derived stem cells	Diabetic rat	Allogeneic	10 mm full-thickness cutaneous wound	Increase wound closure, expression levels of VEGF, p-STAT3, and SDF-1, proliferation of endothelial cells, and neovascularization	–	160×*g* for 20 min/400×*g* for 20 min	Thrombin	[3]
PRP	Adipose-derived stem cells	Diabetic rat	Allogeneic	5 mm full-thickness cutaneous wound	Increase wound closure, reepithelialization and granulation tissue formation, angiogenesis, collagen, epidermal thickness, and EPSC proliferation, downregulate Notch signaling	Citrate phosphate dextrose	160×*g* for 10 min/250×*g* for 15 min	Calcium chloride	[4]
PRP	Sodium alginate hydrogel	Rat	Allogeneic	1.0 cm × 1.0 cm full-thickness excisional wound	Sodium alginate-based PRP hydrogel promote wound healing	–	2800 rpm for 10 min/2800 rpm, 5 min	Thrombin/FeCl3	[83]
PRP	Hyaluronic Acid	Hman	Autologous	Pressure ulcers	Reduce ulcer size and increase healing	sodium citrate	460 g for 8 min	Calcium chloride	[84]
PRP	Fibrin glue and collagen matrix	Human	Allogeneic	Aggressive, refractory, life-threatening wounds	Accelerate and promote wound healing	–	2000×*g* for 2 min/4000×*g* for 8 min	–	[52]
Platelet concentrate	Fibrinogen	Human	Allogeneic	Diabetic foot ulcers	Accelerate and promote wound healing	–	Blood bank platelet concentrate	Thrombin	[53]
PRF^b^	Nanofibrous dressing	Rat	Allogeneic	Full-thickness excisional wound	Accelerated the wound healing, collagen deposition, and the formation of skin appendages	–	2700 rpm for 12 min	–	[85]
PL^c^	Bilayered Fibrin-Based Electrospun-Sprayed Scaffold	Diabetic mice	Xenograft (PRP was for human)	8 mm full-thickness excisional wound	Accelerated wound closure, increase re-epithelialization and collagen deposition	K3EDTA	300 g for 10 min/1500 g for 15 min	–	[86]
PL	Fibrin-based scaffold	Diabetic mice	Xenograft (PRP was for human)	8 mm full-thickness excisional wound	Accelerated wound closure, increased collagen deposition, re-epithelialization, granulation tissue formation	–	300g for 10min/1500g for 15 min	Freeze-thaw/−80/37 °C 3 time	[87]
PL	Poly lactic-co-glycolic acid nanoparticles/hydrogel	Mice	–	Full-thickness cutaneous wound	Increase wound closure	–	–	–	[88]
PL	Collagen/Gelatin Scaffold	Mice	Xenograft (PL/PG was for human)	8 mm full-thickness excisional wound	Accelerated wound healing and enhanced cell proliferation and vessel growth in granulation tissue	–	3000 g for 30 min for remove derbies and filtered the supernatant through a 0.2 μL filter	Freeze-thaw/−80/37 °C	[61]
PL	Gelatin hydrogel	Mice	Xenograft (expired irradiated platelet concentrate)	6 mm full-thickness excisional wound	No significant difference in wound healing was observed between the 3 types of PL used (the cryopreservation PL, the cryopreservation and lyophilization PL, the lyophilization and refrigeration PL)	Heparin sodium	3000 g at 4 C for 30 min and filtered the supernatant through a 0.22 μL filter	Freeze-thaw/−80/37 °C 3 time	[39]
PL	Gelatin Hydrogel	Mice	Xenograft (expired irradiated platelet concentrate)	6 mm full-thickness excisional wound	Accelerated the granulation tissue formation, capillary formation, longest epithelium formation, and promote wound healing process	Heparin sodium	3000 g at 4 C for 30 min and filtered the supernatant through a 0.22 μL filter	Freeze-thaw/−80/37 °C 3 time	[38]
PL	Human umbilical cord mesenchymal stem cells	Pig	–	30 mm full-thickness excisional wound	Increase wound healing, collagen formation, neovascularization, and inflammation, regulated the balance between IL-6/TGFb1 and IL-6/Arg-1, upregulate VEGF-a and TGFb1, Col1, and a-SMA	–	–	–	[89]
PL/Platelet gel	Bioactive dressings	Rat	Xenograft (PL/Platelet gel was for human)	4 mm-full thickness burns (105 °C for 40 s), 6 mm full-thickness excisional wound	Improves the bioactive dressing (Alpha Tocopherol and Ag Sulfadiazine Chitosan Oleate Nanocarriers) effect and PL efficacy on wound healing	–	–	For PL: Freezing −80 °C 5 h/thawing 37 °C For PG: Thrombin/calcium gluconate	[90]
ACS^d^	Gauze dressing (soaked with ACS)	Human	Autologous	Hard-to-heal wound	Decrease wound surface area and promote wound healing	–	15 min at 1500 rpm incubated for 6 h at 37 °C	Glass beads	[12]

˧ Platelet poor plasma.

a Platelet rich plasma.

b Platelet rich fibrin.

c Platelet lysate.

d Autologous conditioned serum.

### 3.2 Blood derivatives compounds in wound healing

**Fig. 3:**
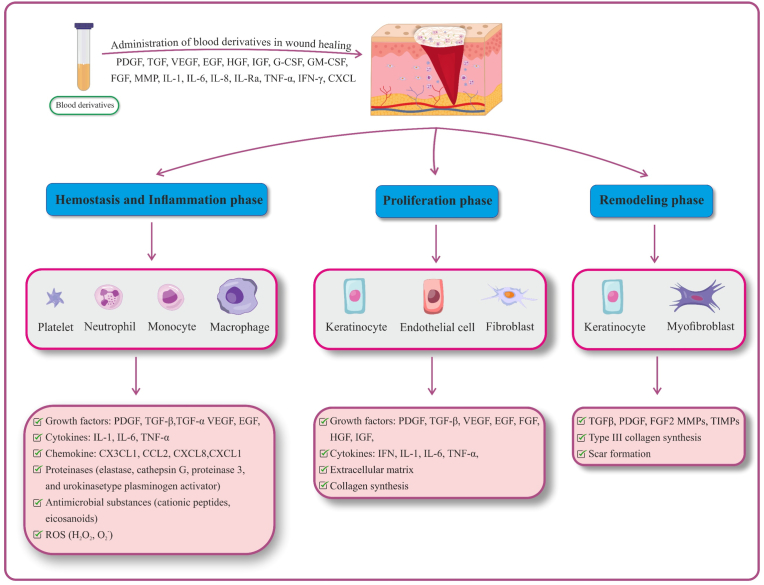
**Roles of blood derivatives in different phases of wound healing.** Growth factors, cytokines, and other biomolecules in blood derivatives promote wound healing by influencing cell proliferation, differentiation, migration, and immunomodulation. PDGF: platelet-derived growth factor, EGF: epidermal growth factor, FGF: fibroblast growth factor, VEGF: vascular endothelial growth factor, IGF: insulinlike growth factor, TGF-β: transforming growth factor-beta, HGF: hepatocyte growth factor, TNF-α: tumor necrosis factor alpha, IL: interleukin, IL-1Ra: interleukin 1 receptor antagonist, GM-CSF: granulocyte macrophage colony-stimulating growth factor, G-CSF: granulocyte colony-stimulating growth factor, MMP: matrix metalloproteinases, TIMP: tissue inhibitor of MMP, ROS: reactive oxygen species.

The focus of wound healing has shifted from macroscopic cellular stages to a molecular understanding of growth factors, directly enabling improved treatments [54]. The function of blood derivatives in wound healing is shown in Fig. 3.

Growth factors and cytokines in blood derivatives regulate processes such as proliferation, differentiation, migration, immunomodulation, re-epithelialization, angiogenesis, matrix formation, remodeling, neurogenesis, and collagen production [5]. Compounds found in blood derivatives contribute to various phases of wound healing. In the hemostasis phase, platelets are activated and initiate wound healing by releasing various growth factors (e.g., PDGF, EGF, TGF-β) and chemotactic signals that promote the transition to the inflammatory phase. Neutrophils, monocytes, and macrophages secrete growth factors and cytokines which contribute to immune reaction and tissue repair. Anti-inflammatory cytokines such as IL-1Ra, IL-4, and IL-13, found in ACS, play crucial roles in modulating inflammation and facilitating wound healing [12,55]. In the proliferative phase, growth factors including bFGF, VEGF, PDGF, and TGF-β promote angiogenesis and enhance keratinocyte migration and proliferation. The final stage, the remodeling phase, involves ECM reorganization and collagen remodeling, characterized by the replacement of type III collagen with type I collagen. These processes are mediated by the signaling pathways of growth factors such as bFGF and TGF-β, as well as the activity of keratinocytes and fibroblasts [[56], [57], [58], [59]].

The concentration of growth factors differs across blood derivatives and is closely linked to platelet content [60]. EGF, one of the first identified wound-healing factors, promotes fibroblast and endothelial proliferation, fibroplasia, angiogenesis, and collagen activity [54]. However, growth factors rarely act alone and their effects depend on concentration and interaction with other mediators. While physiological levels of growth factors promote wound healing, excessively high concentrations of growth factors can impair cells proliferation and wound closure [61]. On the other hand, the angiogenic function of VEGF is amplified when co-administered with FGF, EGF, IGF-1, TGF-β, and PDGF [61,62]. Angiogenesis plays a critical role in wound healing, especially in conditions such as diabetic foot ulcers, where it is often compromised [3,4]. Growth factors and cytokines such as TGF-β, VEGF, EGF, FGF, IGF, and IL-1β are key regulators of angiogenesis [5,63], and numerous studies have confirmed that blood derivatives enhance angiogenesis in wound healing [5]. Collagen deposition and re-epithelialization are also enhanced by blood derivatives. Growth factors such as TGF-β, VEGF, PDGF, EGF, and IGF regulate these processes, improving wound outcomes [3,4,64].

Blood derivatives are suitable for long-term storage without significant loss of activity. Freezing at −80 °C is common, and lyophilization further enhances stability. Platelet lysate–derived growth factors, for instance, remain stable at 4 °C for up to nine months [38], making clinical application more feasible.

## 4 Future and prospective

Blood derivatives have garnered increasing interest among researchers and clinicians due to their regenerative potential. PRP emerged as the first generation of platelet concentrates [6], followed by PRF, representing the second generation [6,42]. Other blood products, such as PL and ACS, have also gained attention. PL, like PRP, contains high levels of growth factors and has been used in both regenerative medicine and the treatment of various conditions [5]. ACS, which is rich in anti-inflammatory cytokines, especially IL-1Ra, was initially applied in the treatment of osteoarthritis [35]; but its use has expanded to include wound healing applications [12,65]. To improve the application of blood products, techniques have been developed to convert them into gel formulations. Their combination with other therapeutic strategies has further expanded their clinical use and enhanced treatment efficacy. Future directions may involve wider adoption of these products. For example, lyophilized PL has demonstrated long-term stability at 4 °C [38], and incorporating such powders into wound dressings may offer a simple, cost-effective solution for wound care. Innovations may also include compounds that activate blood cells to increase the release of growth factors and anti-inflammatory cytokines. Given the ease of preparation and the possibility of autologous or allogeneic use, blood derivatives are expected to occupy an increasingly prominent role in regenerative medicine, particularly in wound healing.

## 5 Conclusion

The growing diversity of blood derivatives has attracted significant attention in recent years and has led to their expanded use in wound healing. Products such as PRP, PRF, PL, and ACS have demonstrated considerable efficacy in both alone and combination therapy with other treatments like hydrogels and stem cells. Additionally, the ability to apply these products in both liquid and gel forms has broadened their practical applications.

Nevertheless, further comparative studies are needed to evaluate the relative effectiveness of different blood derivatives. The development of a novel product that combines the benefits of multiple blood-derived components may offer patients more effective and tailored treatment options.

## Data availability statement

Not applicable.

## Ethics approval

Not applicable.

## Authors’ contributions

All authors contributed to the work's conception and main idea. M.Z drafted the main text, figures, and tables. M.M and F.P reviewed and revised the text. M.M.H supervised the work and provided comments and additional scientific information. All authors read and approved the final version of the work to be published.

**Table:** Abbreviation list

Abbreviation	Definition
ACS	Autologous conditioned serum
b-FGF	basic-fibroblast growth factor
ECM	Extracellular matrix
EGF	Epidermal growth factor
EPC	Endothelial progenitor cell
FBS	fetal bovine serum
G-CSF	Granulocyte colony-stimulating growth factor
GM-CSF	Granulocyte macrophage colony-stimulating growth factor
HB-EGF	heparin-binding EGF-like growth factor
HGF	Hepatocyte growth factor
IGF	Insulin-like growth factor
IL	Interleukin
IL-1Ra	Interleukin 1 receptor antagonist
KGF	Keratinocyte growth factor
MMP	Matrix metalloproteinase
PDGF	Platelet-derived growth factor
PPP	Platelet poor plasma
PL	Platelet lysate
PRF	Platelet rich fibrin
PRP	Platelet rich plasma
ROS	Reactive oxygen species
TIMP	Tissue inhibitor of MMP
TGF-β	Transforming growth factor beta
TGF-α	Transforming growth factor alpha
TNF-α	Tumor necrosis factor alpha
VEGF	Vascular endothelial growth factor

## Funding

Not applicable.

## Declaration of competing interest

The authors declare no conflict of interest.
